# Chromosome-Level Genome Assembly of Red Sea Bream (*Pagrus major*) Reveals Integration of Heterospecific Sperm-Derived Genetic Material in Artificial Gynogenesis

**DOI:** 10.3390/biom15121648

**Published:** 2025-11-24

**Authors:** Mingyang Liu, Guixing Wang, Yuqin Ren, Xiaoyan Zhang, Bingbu Li, Yitong Zhang, Yucong Yang, Lize San, Jilun Hou

**Affiliations:** 1 State Key Laboratory of Mariculture Biobreeding and Sustainable Goods, Beidaihe Central Experiment Station, Chinese Academy of Fishery Sciences, Qinhuangdao 066100, China; liumy@bces.ac.cn (M.L.); wanggx@bces.ac.cn (G.W.); renyq@bces.ac.cn (Y.R.); zhangxy@bces.ac.cn (X.Z.); libb@bces.ac.cn (B.L.); zhangyt@bces.ac.cn (Y.Z.); yangyc@bces.ac.cn (Y.Y.); 2 Hebei Key Laboratory of the Bohai Sea Fish Germplasm Resources Conservation and Utilization, Beidaihe Central Experiment Station, Chinese Academy of Fishery Sciences, Qinhuangdao 066100, China; 3 Bohai Sea Fishery Research Center, Chinese Academy of Fishery Sciences, Qinhuangdao 066100, China

**Keywords:** *Pagrus major*, allo-sperm effect, genome, paternal-specific sequences, gene family expansion, centromeric satellite DNA, spermiogenesis

## Abstract

Artificially induced gynogenesis, a technique that utilizes UV-irradiated sperm to activate eggs while excluding paternal genetic contribution, has been instrumental in the genetic improvement of aquaculture species. Although the allo-sperm effect has been observed in some freshwater fish and suggests the integration of paternal DNA, its occurrence and mechanisms in marine fish remain unclear. In this study, a 795.23 Mb chromosome-level genome assembly for red sea bream (*Pagrus major*) was presented, with a scaffold N50 of 32.03 Mb, encompassing 29,083 protein-coding genes. Furthermore, the allo-sperm effect was investigated on the artificial gynogenesis of Japanese flounder (*Paralichthys olivaceus*) induced by UV-irradiated *P. major* sperm. Whole-genome sequencing of gynogenetic and normal fertilized offspring revealed eight representative genomic sequences with >96.88% nucleotide identity to *P. major*, including six Sparidae-specific centromeric satellite DNA sequences. PCR validation and Sanger sequencing confirmed that these sequences were present exclusively in gynogenetic groups and absent in normally fertilized offspring, providing direct evidence of the allo-sperm effect. Our findings extend the allo-sperm effect to marine fish and demonstrate its potential across taxonomically distant taxa, *P. olivaceus* (Pleuronectiformes) × *P. major* (Spariformes). These results offer valuable genomic information for *P. major*, and provide important insights for future genetic breeding programs in aquaculture.

## 1. Introduction

While sexual reproduction remains the dominant reproductive strategy in vertebrates, asexual strategies such as parthenogenesis and gynogenesis persist in certain groups, including fish, amphibians, and reptiles [1]. Natural gynogenesis, a form of asexual reproduction characterized by sperm-dependent embryogenesis without incorporating paternal genetic material, has been documented in several fish species [2]. Artificial gynogenesis was first established in the loach (*Misgurnus anguillicaudatus*) [3] and has since been successfully extended to a wide range of economical aquaculture species, including half-smooth tongue sole (*Cynoglossus semilaevis*) [4], Croceine croaker (*Pseudosciaena crocea*) [5], and grass carp (*Ctenopharyngodon idella*) [6], making it an effective tool for genome homozygosis, sex control, and genetic analysis [7,8].

In UV-irradiated sperm, paternal DNA is fragmented, while still triggering egg development, resulting in offspring that retain only maternal genetic material. However, gynogenetic offspring may exhibit paternal traits, indicating the integration of paternal DNA, referred to as the allo-sperm effect [5]. By using diverse paternal sperm to induce gynogenesis, offspring may exhibit paternal-specific genotypes and phenotypes following the allo-sperm effect [9]. Heterologous paternal DNA fragments have been detected in gynogenetic offspring, providing further evidence of this phenomenon [10,11]. In gynogenetic grass carp, a paternal HoxC6b fragment and its recombinant derivative from koi carp sperm were recovered, indicating stable incorporation of paternal DNA into gynogenetic progeny [11]. In gibel carp clone F, 12 paternal DNA fragments derived from blunt snout bream were retained over 13 successive gynogenetic generations, indicating chromosomal integration of paternal sequences with CgA22_34 stably inserted into one of the three homologous chromosomes [10]. While the allo-sperm effect has been documented in freshwater fish, its presence in marine species remains to be fully investigated. Such studies would expand our understanding of this phenomenon across aquatic environments.

Chromosome-level reference genome is fundamental for dissecting species evolution, trait determination, and aberrant reproductive mechanisms. By unambiguously anchoring contigs along entire chromosomes, it dramatically improves the continuity and accuracy of centromeres, telomeres, and repetitive regions, ensuring that structural-variant detection, selective-signal identification, and linkage analyses are no longer confounded by assembly fragmentation. Red sea bream (*Pagrus major*) is a commercially important marine species in East Asia [12,13]. The sperm of *P. major* was used as a heterologous sperm to artificially induce gynogenesis in Japanese flounder (*Paralichthys olivaceus*) due to its similar size and motility [6,14]. Gynogenetic *P. olivaceus* induced with heterologous sperm of *P. major* may undergo the integration of paternal DNA fragments [7]. In this study, we present a high-quality chromosome-level genome assembly of *P. major* (2n = 48) and use it to investigate the unique genes involved in fish spermatogenesis through gene-family clustering and phylogenomic reconstruction. The study further revealed potential paternal DNA integration in gynogenetic offspring of *P. olivaceus* by a whole-genome sequencing analysis. These findings offer novel insights into the genetic basis of gynogenesis and the mechanisms underlying the allo-sperm effect in marine fish.

## 2. Materials and Methods

### 2.1. Preparation of Experimental Animals

**Figure 1:**
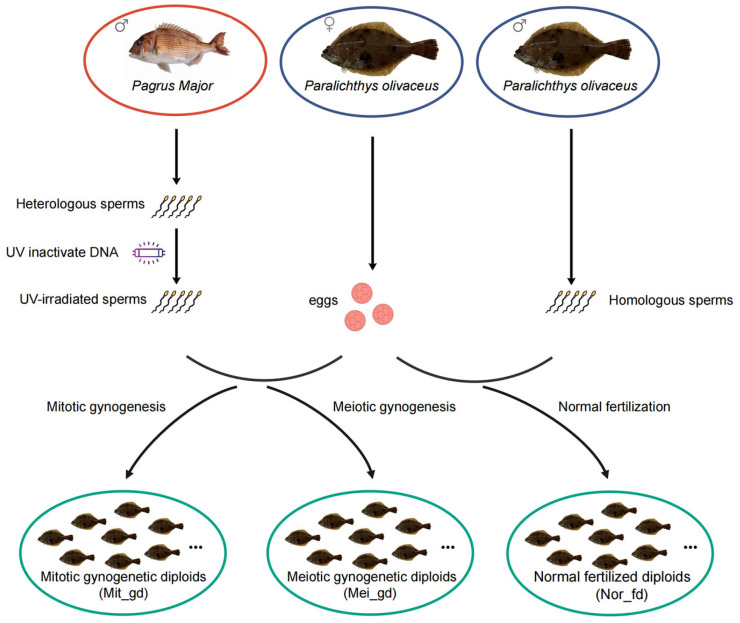
Overview of the breeding design.

Sexually mature *P. olivaceus* (one female and one male) and *P. major* (one male) with well-developed gonads and normal external morphology were selected as broodstock from the Beidaihe Experimental Station, Chinese Academy of Fishery Sciences (CAFS), China. Eggs obtained from female *P. olivaceus* were evenly divided into three experimental groups. Both groups underwent gynogenesis using UV-irradiated *P. major* sperm at a dose of 50 mJ/cm^2^ and protocols for inducing mitotic and meiotic gynogenesis were adapted from a previous study [6,14]. Meiotic gynogenesis (Mei_gd) was induced by cold shock (0 °C for 45 min), administered 3 min post-fertilization at 17 °C to suppress 2nd polar body extrusion. Mitotic gynogenesis (Mit_gd) was induced by applying hydrostatic pressure (650 kg/cm^2^ for 6 min) during the first mitotic metaphase to inhibit nuclear division (Figure 1). The third group served as a diploid control (Nor_fd) and was produced through normal fertilization with *P. olivaceus* sperm. Fertilized eggs from the gynogenetic and control groups were separately incubated in 300-L circular tanks under static water conditions with continuous aeration, maintaining a temperature regime of 17–18 °C. Fry were initially fed rotifers until day 20, after which the system was converted to recirculating water, and fry were fed *Artemia* nauplii until metamorphosis.

### 2.2. Sample Collection, DNA and RNA Extraction

From *P. olivaceus* samples, a total of 90 healthy fry (30 days old) were randomly collected, with 30 from each group (Mit_gd, Mei_gd and Nor_fd). Muscle tissue was sampled and flash-frozen in liquid nitrogen. Genomic DNA was extracted from approximately 200 mg of tissue using the TIANamp Marine Animal DNA Kit (DP324, TIANGEN, Beijing, China) following the manufacturer’s protocol. DNA integrity was assessed via 1% agarose gel electrophoresis, and quantification and purity assessment (A260/A280 ratios) were performed using a NanoDrop 2000 (Thermo Fisher Scientific, Waltham, MA, USA).

From *P. major*, one sexually mature male was selected for tissue collection. Thirteen tissues (stomach, gill, liver, kidney, spleen, intestine, fin, heart, eyes, skin, brain, muscle, and gonads) were aseptically dissected and immediately frozen in liquid nitrogen for preservation. DNA was extracted using the TIANamp Marine Animal DNA Kit (DP324, TIANGEN, Beijing, China) according to the manufacturer’s instructions. Total RNA was isolated using TRIzol reagent (15596018CN, Invitrogen, Carlsbad, CA, USA), and quality was verified via NanoDrop 2000 and 1% agarose gel electrophoresis.

### 2.3. Whole Genome Sequencing, Library Construction, Sequencing, and Genome Survey

Whole-genome sequencing (WGS) libraries were constructed using 0.5 μg of genomic DNA. Paired-end sequencing (2 × 150 bp) was performed using the Illumina NovaSeq 6000 platform. Raw reads were filtered using Fastp v0.23.4 [15] to remove adapter sequences and low-quality reads. Genome characteristics were analyzed through k-mer frequency distribution (k = 21) using Jellyfish v2.3.0 [16], with subsequent estimations of genome size, heterozygosity, repetitive element content, and GC composition via GenomeScope2 v1.0.0 [17].

### 2.4. PacBio Library Construction, Sequencing, and De Novo Assembly

Genomic DNA libraries with an average insert size of 20 kb were constructed using the SMRTbell Express Template Prep Kit 2.0 (100-938-900; PacBio Biosciences, Menlo Park, CA, USA) and sequenced on the PacBio Sequel II platform (Pacific Biosciences, Menlo Park, CA, USA) in the circular consensus sequencing (CCS) mode, generating HiFi reads with min-passes = 3 and min-rq = 0.99. CCS reads were generated using the SMRT Link [18] and assembled into contigs using Hifiasm v0.19.6 [19,20] with default parameters.

### 2.5. Hi-C Library Preparation, Sequencing, and Chromosome Anchoring

A Hi-C library was constructed using 1 μg of genomic DNA of *P. major* and sequenced on the Illumina NovaSeq 6000 platform (paired-end 150 bp). Raw sequencing data were processed using the Juicer v1.6 [21] to generate matrices and perform bias correction. Subsequently, Contigs were anchored to 24 pseudochromosomes using 3D-DNA v180922 [22], and manually curated with Juicebox Assembly Tools v1.9.1 [21] to rectify misplacements and generate a chromosome-scale assembly.

### 2.6. Gene Annotation

Repetitive elements were annotated by de novo homology-based methods. RepeatModeler2 v2.0.5 [23] and LTR_FINDER v1.0.7 [24] were used for de novo library construction to systematically identify interspersed repeats and long terminal repeat (LTR) retrotransposons. Subsequently, RepeatMasker v4.0.9 [25] was used for comprehensive repeat classification and masking, incorporating both the de novo library and RepBase database for homology-based detection.

Protein-coding gene prediction combined three strategies: ab initio, homology-based, and transcriptome evidence-based prediction. For transcriptome prediction, RNA isolated from 13 tissues was pooled and sequenced using the PacBio CCS platform. Full-length transcripts were processed using the IsoSeq v3.4.0 pipeline (https://github.com/PacificBiosciences/IsoSeq, accessed on 14 May 2024) with parameters—min-passes 1—min-rq 0.95 to obtain high-quality CCS. Transcript assembly was performed using StringTie v1.2.3 [26], and the resulting isoforms were aligned to the genome using the Program to Assemble Spliced Alignments (PASA) v2.4.1 [27] to demarcate exon-intron boundaries. To ensure completeness, TransDecoder v5.1.0 (http://transdecoder.sourceforge.net/, accessed on 14 May 2024) was used to filter protein-coding sequences. Homology-based prediction utilized miniprot [28] to align protein sequences from two closely related species (*Sparus aurata* and *Acanthopagrus latus*) against the genome for protein evidence inference. For ab initio prediction, Augustus v3.4.0 [29] was implemented with iterative training using transcriptome and homology evidence to optimize the gene model parameters. Further, EVidenceModeler v2.1.0 [30] was employed to integrate the three types of evidence. Final gene structure predictions were functionally annotated using EggNOG, SwissProt [31], TrEMBL [32], InterPro [33], and NCBI NR databases.

### 2.7. Genome Assembly and Annotation Evaluation

Assembly completeness was assessed using Benchmarking Universal Single-Copy Orthologs (BUSCO) v4.1 [34] with the Actinopterygii_odb10 lineage-specific database. Genome assembly metrics, including size and N50 statistics, were evaluated using QUAST v5.0.2 [35]. The GFF3 annotation files for the longest transcripts of the protein-coding genes were extracted, and gene annotation information was compiled using Gtftk v1.0 [36].

### 2.8. Comparative Genomic Analyses

The orthogroups (OGs) were identified by comparing the predicted protein sequences of *P. major* with 12 other species using OrthoFinder v2.5.4 [37] under default parameters. Single-copy orthogroups were aligned using MUSCLE v3.8.31 [38] with the default settings. A maximum-likelihood species tree was inferred from the concatenated alignment of single-copy orthologous proteins using IQ-TREE v2.2.0 [39]. Divergence times were estimated using single-copy orthologs by MCMCTREE in the PAML v4.9 package [40], and calibrated with fossil divergence times obtained from the TimeTree database (http://www.timetree.org/, access on 25 June 2024) [41]. Gene family expansion and contraction were assessed using CAFE5 v1.1 [42], and functional enrichment was assessed using KEGG pathway mapping [43] and Fisher’s exact test (*p* < 0.05). To investigate the evolutionary development of piscine sperm morphology, we constructed a phylogenetic tree using 12 teleost species with well-documented sperm ultrastructural data. These species represent the Sparidae [44,45], Tetraodontidae [46], Pleuronectiformes [47,48,49], Salmoniformes [50,51], Cypriniformes [52], Characiformes [53], Perciformes [54], and Siluriformes [55] (Table A5).

### 2.9. WGS Quality Control and Alignment

Raw reads were filtered by Fastp v0.23.4 [15], removing adaptor sequences, Poly-N, and low-quality reads (Q ≤ 5). Reference genome indices for *P. olivaceus* [56] and *P. major* were constructed. Reads were aligned using BWA v0.7.17 [57]. SAM files were converted to BAM format using Samtools v1.1.9 [58]. Paternal-specific reads were extracted using a two-step filtering process: (1) reads unmapped to *P. olivaceus*, and (2) reads successfully mapped to *P. major*.

### 2.10. Detection of Paternal-Specific Sequences in Gynogenetic Offspring

Paternal-specific reads were independently assembled using SOAPdenovo2 v.242 [59], SPAdes v4.0.0 [60], and MEGAHIT v1.2.9 [61]. Stringent filtering was applied to define authentic paternal sequences. A custom analytical pipeline incorporating multi-layered filtering was developed: (1) Fragment presence was required in ≥3 gynogenetic offspring; (2) Strict criteria ensured paternal-specific sequences were present only in the gynogenetic offspring and absent in the offspring from normal fertilization; (3) Sequence length thresholds (>200 bp) were enforced using SeqKit v2.8.1 [62] for quality control; (4) CD-HIT v4.8.1 [63] clustering (90% identity) removed redundant sequences while preserving representative fragments.

### 2.11. PCR Validation

**Figure A1:**
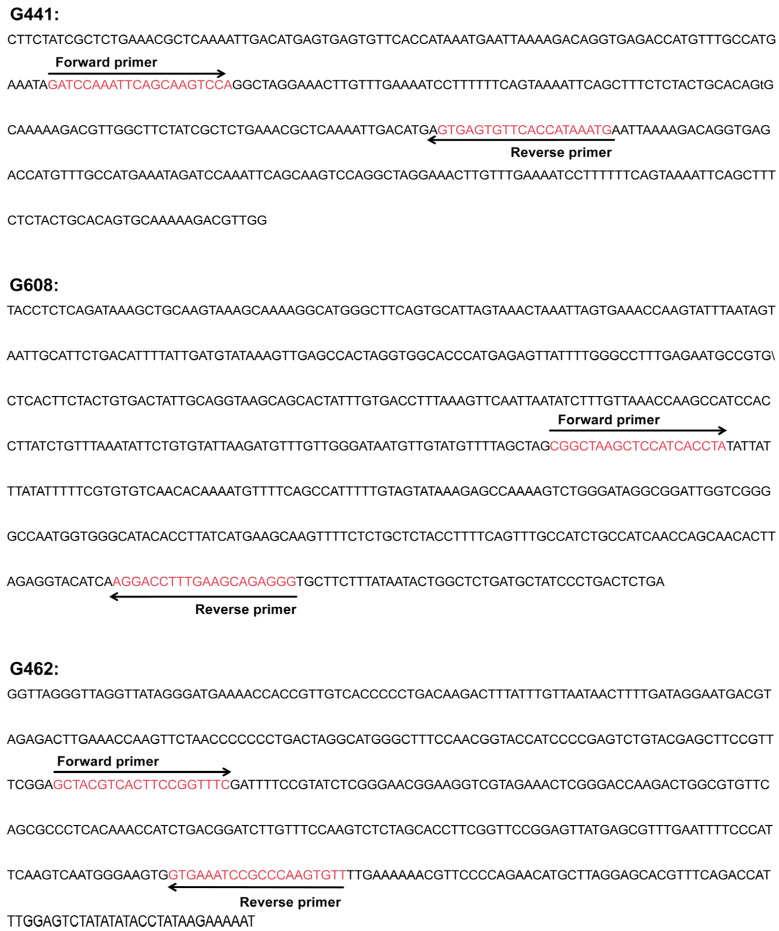
Primer-map schematics for G441, G608 and G462.

PCR validation of paternal-specific sequences was conducted on three experimental groups (Mit_gd, Met_gd, and Nor_fd), two additional groups (ad-Mit_gd and ad-Met_gd), and *P. major*. Paternal-specific primers (Table A1) (Figure A1) were used for PCR amplification on an A300 instrument (LongGene, Hangzhou, China). The reaction protocol comprised: initial denaturation at 95 °C for 3 min; 35 amplification cycles of 94 °C for 20 s, 58 °C for 20 s, 72 for 40 s; and final extension at 72 °C for 5 min. PCR amplification products were detected by 1.5% agarose gel electrophoresis (100 V, 45 min).

### 2.12. Sanger Sequencing

Total DNA extracted from Mit_gd, Met_gd, and Nor_fd was used as the template for PCR amplification, and three randomly chosen samples from each group were subjected to Sanger sequencing. The target band was excised from the gel and purified using the TIANgel Midi Purification Kit (DP209, TIANGEN, Beijing, China). The Gel-purified amplicons were cloned into the Hieff Clone^®^ Universal Zero TOPO TA/Cloning kit (10906ES08, YEASEN, Shanghai, China), and plasmids from positive clones were isolated using the TIANprep Mini Plasmid Kit (DP103, TIANGEN, Beijing, China) for Sanger sequencing. The EcoRI centromeric satellite DNA was identified and annotated with SnapGene v4.3.11 (www.snapgene.com, access on 7 November 2025).

## 3. Results

### 3.1. Genome Assembly

**Figure 2:**
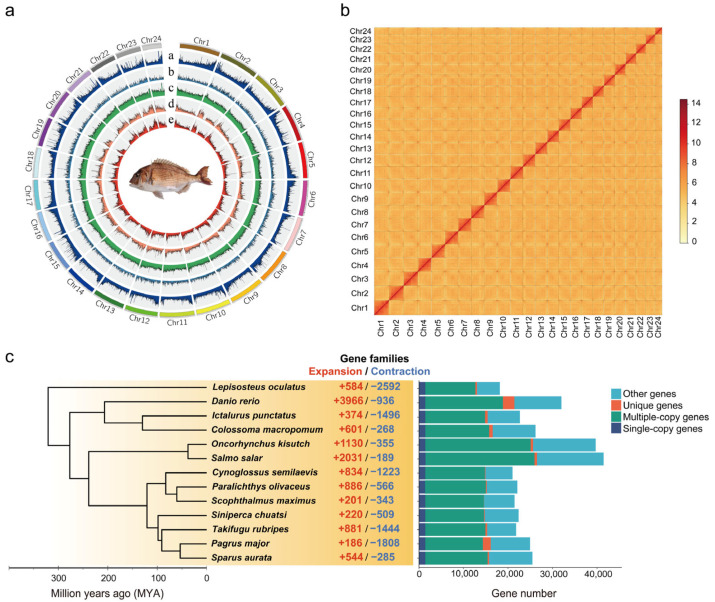
High-quality genome assembly of the *P. major* and genome evaluation. (**a**) Global view of *P. major*: a. SNPs density; b. InDels density; c. GC contents; d. gene density; e. Repetitive element density; (**b**) Genome-wide chromatin interactions of 24 chromosomes. (**c**) The rooted phylogenetic tree (Cladogram) of 13 fish species reconstructs their evolutionary relationships. The central panel reflects the number of expanded and contracted gene families. The right panel summarizes the counts of orthologous genes classified as single-copy, multi-copy, unique, and other categories.

To generate a chromosome-level genome of *P. major*, high-depth sequencing was performed on muscle tissue using Illumina short reads and PacBio HiFi long reads (Table 1). A total of 43.09 Gb (~55.8×) of Illumina raw data underwent genome survey, revealing an estimated genome size of approximately 772.32 Mb with a heterozygosity of 0.65% based on K-mer frequency analysis (Table 1) (Figure 2a). Further, the de novo assembly of PacBio HiFi reads yielded a genome assembly size of 795.23 Mb, with 362 contigs and a contig N50 of 11.22 Mb (Table 1). A total of 24 pseudochromosomes were successfully anchored, with chromosome numbers consistent with those previously reported for other Sparidae species (Table 1) (Figure 2b). The final assembly had a scaffold N50 of 32.03 Mb and an anchoring rate of 95.44% (Table 1). The BUSCO analysis reveals identification of 97.8% in the actinopterygii_odb10 database, indicating high completeness.

### 3.2. Genome Annotation

Repeat analysis revealed 246.23 Mb of repetitive elements, representing 30.96% of the genome. These included Class II DNA transposons (9.20%) and Class I retrotransposons, including LINEs (4.54%), SINEs (0.32%), and LTRs (2.43%), along with 10.72% unclassified repeats (Table A2). A total of 29,083 protein-coding genes were annotated using a combined strategy of transcriptome evidence, ab initio prediction, and homology-based methods. The gene structures showed considerable complexity, with average gene (13,821.71 bp), exon (183.32 bp), and intron (1566.45 bp) lengths exceeding the teleost averages. BUSCO analysis of annotated proteins recovered 93.6% of actinopterygii_odb10 orthologs. Comparative annotation against related species, such as *S. aurata*, *A. Latus*, *P. olivaceus* and *Takifugu rubripes*, demonstrating high concordance (Table A3). Functional annotation was achieved for 27,474 genes (94.47%) using TrEMBL, NR, SwissProt, InterPro, and EggNOG databases (Table A4).

### 3.3. Phylogenetic Analysis and Gene Family Expansion

**Figure A2:**
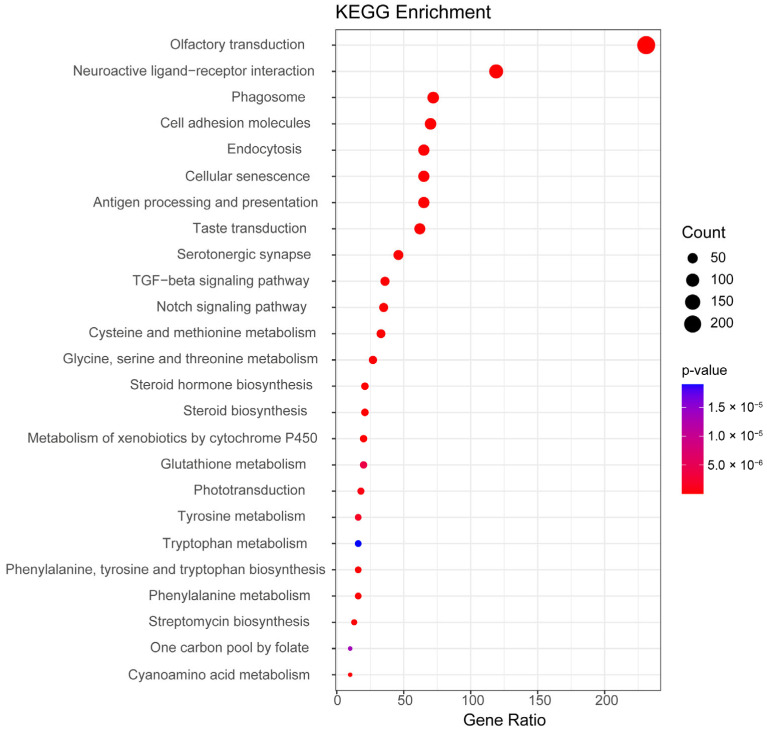
Top-25 KEGG Pathway Enrichment Results for Expanded Gene Families in Clades with Smaller Sperm Morphology.

The molecular phylogeny revealed that *P. major* and *S. aurata* form a sister clade, with a divergence time of ~52.98 million years ago (MYA), consistent with their taxonomic classification within the Sparidae family. Notably, a monophyletic group consisting of *P. major*, *S. aurata*, *T. rubripes*, *Siniperca chuatsi*, *Scophthalmus maximus*, *P. olivaceus*, and *Cynoglossus semilaevis* diverged from the Salmoniformes (*Salmo salar* and *Oncorhynchus kisutch*) around 238.48 MYA, a period corresponding with significant diversification in sperm ultrastructure dimensions (Figure 2c). Comparative genomics analysis identified 75 gene families (comprising 2352 genes) that were significantly expanded in the clade characterized by smaller sperm morphology (Figure 2c). These expanded gene families showed significant enrichment (*p* < 0.05) in 36 KEGG pathways, including Olfactory transduction (ko04740), Notch signaling (ko04330), TGF-beta signaling (ko04350), steroid biosynthesis (ko00100), carbohydrate digestion and absorption (ko04973), and cellular senescence (ko04218). These findings suggest a potential regulatory role for these pathways in spermiogenesis and sperm structural specialization (Figure A2).

### 3.4. Whole Genome Sequencing and Alignment

To examine the paternal contribution to artificial gynogenesis, WGS was performed on 30 offspring samples from three groups: Mit_gd, Mei_gd, and Nor_fd. A total of 604.71 Gb of raw sequencing data was generated, with 599.50 Gb of clean data retained after quality control (average 6.66 Gb per sample, mean depth 11.32×). Initial alignment to the *P. olivaceus* reference genome showed high mapping rates across all groups: 99.57 ± 0.22% for Mit_gd, 99.69 ± 0.04% for Mei_gd, and 99.63 ± 0.07% for Nor_fd (Table 2). To assess potential paternal genetic contributions, the unmapped reads were subsequently aligned to the chromosome-level genome of *P. major*. The alignment rates to *P. major* were 20.48 ± 12.65% for Mit_gd, 18.60 ± 3.00% for Mei_gd, and 12.12 ± 2.03% for Nor_fd. A chi-squared test revealed a statistically significant difference in alignment rates between Mit_gd and Nor_fd (*p*-value = 0.00001), suggesting a possible paternal influence (Table 2).

### 3.5. Detection of Paternal-Specific Sequences in Offspring of Gynogenesis

Paternal-specific sequences from Mit_gd, Mei_gd, and Nor_fd were subjected to de novo assembly using three distinct software tools—SOAPdenovo2, SPAdes, and MEGAHIT (Table A6). Among them, MEGAHIT demonstrated superior assembly continuity, as reflected by its higher N50 values compared to SOAPdenovo2 and SPAdes (524.27 bp vs. 456.97 and 484.93 bp, respectively). The MEGAHIT assemblies yielded average total lengths of 95.85 kb (Mit_gd), 47.47 kb (Mei_gd), and 41.81 kb (Nor_fd), with corresponding average N50 values of 524.27 bp, 456.97 bp, and 484.93 bp. The average number of contigs per assembly was 183.90, 111.70, and 91.87 respectively. To rigorously identify paternal-specific sequences, a custom filtering pipeline with strict inclusion criteria was implemented. Only sequences present in gynogenetic offspring but absent in normally fertilized progeny were retained. This stringent approach identified 37 (0.33%), 182 (3.78%), and 15 (0.25%) paternal-specific sequences from the SOAPdenovo2, SPAdes, and MEGAHIT assemblies, respectively.

**Figure 3:**
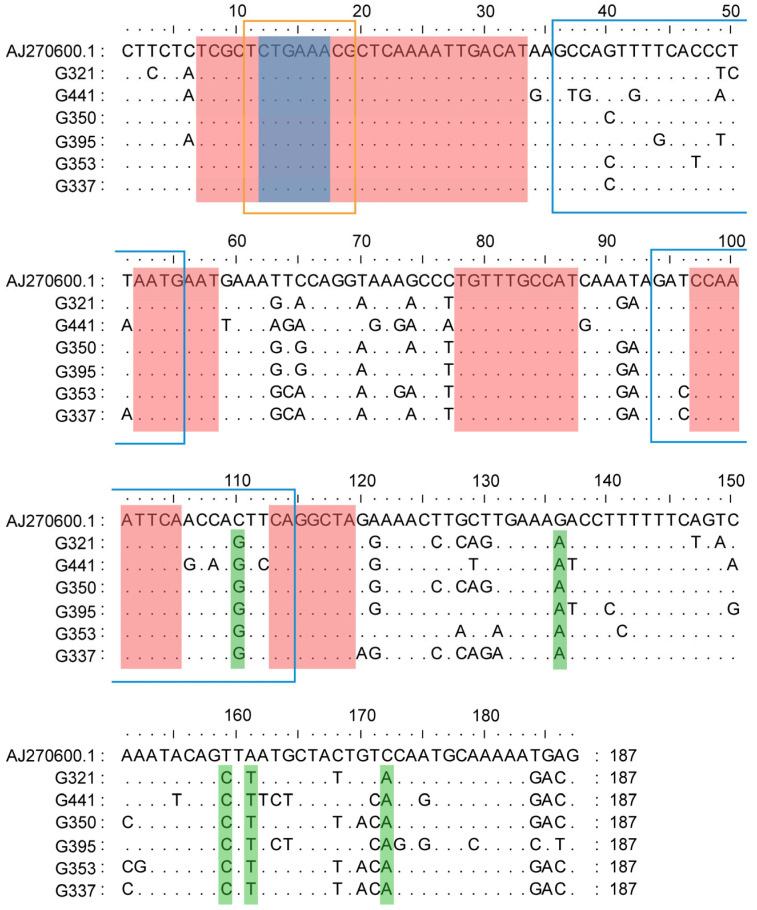
Alignment of paternal-specific sequences (G321, G441, G350, G395, G353, and G337) with the reference sequence AJ270600.1. Blue boxes highlight the PCR Primer. Red shading indicates regions where the paternal-specific sequences share sequence identity with AJ270600.1 exceeding 7 bp. Green shading highlights areas of sequence variation between the paternal-specific sequences and AJ270600.1. The consensus motif (A/T)CTGAAA(A/C)(G/C) is marked with orange boxes, while conserved sequences corresponding to the Sparidae family EcoRI centromeric satellite DNA are shown in blue shading.

**Figure 4:**
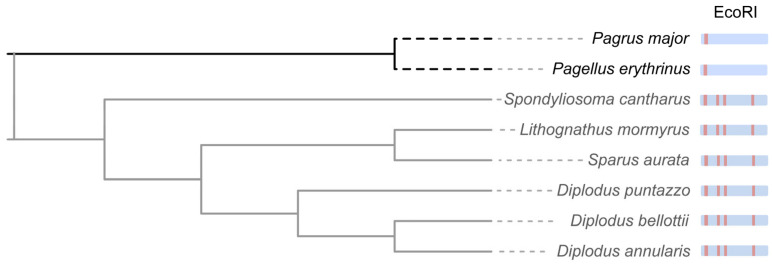
Rooted phylogenetic tree of the Sparidae family with the EcoRI centromeric satellite alignment. Blue shading denotes the 187 bp EcoRI centromeric satellite repeat. Red shading indicates the position of the conserved TCTGAAACG motif.

The 234 candidate sequences underwent redundancy reduction using CD-HIT, which yielded eight representative sequences (338–608 bp). Alignment against the *P. major* genome revealed complete query coverage (100%) and high nucleotide identity (>96.88%) for all representative sequences. Notably, six sequences (G321, G441, G350, G395, G353, and G337) showed exclusive homology to Sparidae family EcoRI centromeric satellite DNA (187 bp tandem repeats), as documented in the NCBI nt database and exhibited a tandem organization. Multiple-segment alignment with the *Dentex gibbosus* centromeric satellite DNA (AJ270600.1) identified a conserved TCTGAAACG motif at positions 11–19, consistent with the characteristic EcoRI satellite structure reported in Sparidae [64] (Figure 3). Phylogenetic relationships among Sparidae species based on EcoRI centromeric satellite DNA demonstrated a close evolutionary relationship between the *Pagrus* and *Pagellus* lineages within the Sparidae family (Figure 4). Notably, both *Pagrus* and *Pagellus* share an identical EcoRI satellite structure, characterized by the presence of the conserved TCTGAAACG motif exclusively at positions 11–19. In contrast, the remaining lineages exhibit this motif at four distinct positions:11–19, 47–55, 68–76, and 147–155, highlighting a unique structural simplification in the *Pagrus* and *Pagellus* clade (Figure 4) [64]. The remaining two sequences (G608 and G402) did not contain the 187-bp repetitive structure.

### 3.6. PCR Detection of Paternal-Specific Sequences

**Figure 5:**
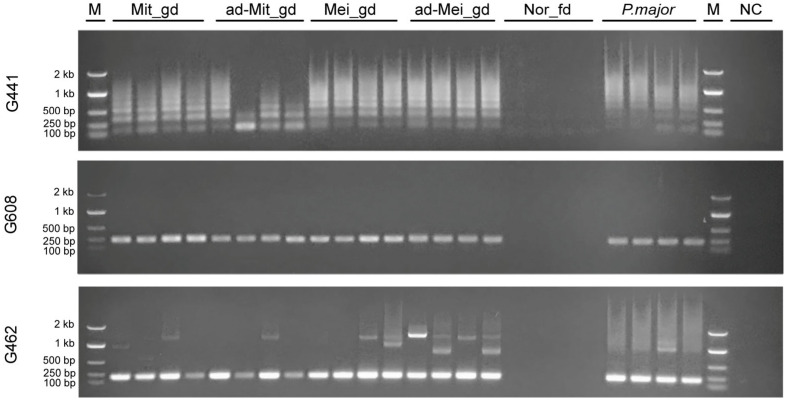
Amplification results for G441, G608, and G462 in Mit_gd, ad-Mit_gd, Mei_gd, ad-Mei_gd, Nor_fd, and *P. major*. M: 2 kb DNA ladder. NC: negative control.

Given that sequences G321, G441, G350, G395, G353, and G337 exhibited high homology with the EcoRI centromeric satellite DNA of the Sparidae family, three primers (G441, G608, and G462) were designed to evaluate the accuracy of the allo-sperm effect. Random samples (*n* = 4 per group) from experimental populations (Mei_gd, Mit_gd, and Nor_fd), the *P. major* population, and additional gynogenetic groups (ad-Mei_gd and ad-Mit_gd) were subjected to PCR amplification. Notably, no electrophoretic bands were detected in the Nor_fd group for any of the three primers. In contrast, amplification products were consistently observed in all gynogenetic groups (Mei_gd, Mit_gd, ad-Mei_gd, and ad-Mit_gd) as well as in the *P. major* population, confirming the presence of paternal genomic contributions (Figure 5). The amplification products from G441-F/R primers displayed tandem repeat structures in both mitotic and meiotic gynogenic populations and in the *P. major* population. These repeat motifs showed structural stability in the meiotically derived groups (Mei_gd and ad-Mei_gd), while variable repeat numbers were observed in the mitotically derived groups (Mit_gd and ad-Mit_gd).

### 3.7. Sanger Sequencing of EcoRI Centromeric Satellite DNA

**Figure 6:**
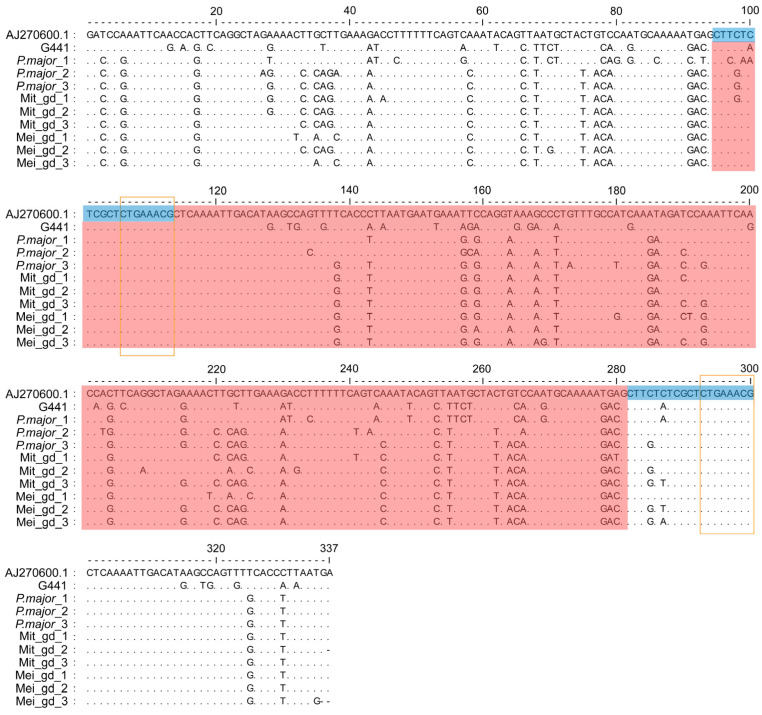
Nucleotide sequence of *P. major* and the gynogenetic groups. Red shading indicates the full-length 187 bp EcoRI centromeric satellite DNA. Blue shading highlights the initial sequence of EcoRI centromeric satellite DNA. The consensus motif (A/T)CTGAAA(A/C)(G/C) is marked with orange boxes.

The Sanger sequencing of the G441 amplicons was performed in Mit_gd, Mei_gd, and *P. major* group, confirming that the EcoRI sequence of *P. major* is indeed present as paternally inherited DNA in gynogenetic *P. olivaceus* (Supplementary File S1). Given the EcoRI sequencing comprises a 187 bp tandem repeat, whereas the PCR product is only 150 bp, the second electrophoretic bands (~337 bp) were excised and subjected to Sanger sequencing to ensure the entire 187 bp EcoRI fragment was captured. The amplications exhibited a distinct 187 bp centromeric repeat architecture that precisely matched G441, and the consensus motif (A/T)CTGAAA(A/C)(G/C) was conserved in both *P. major* and the gynogenetic groups (Figure 6).

## 4. Discussion

High-quality reference genomes are essential for advancing genomic breeding programs and dissecting the genetic basis of complex traits [65]. Extensive genetic and genomic resources have been accumulated and are routinely exploited for constructing high-density linkage maps, pan-genomes, and genome-wide SNP identification [66,67]. Although two genome assemblies (Pmaj_1.0: https://ftp.ncbi.nlm.nih.gov/genomes/all/GCA/002/897/255/GCA_002897255.1_Pmaj_1.0/, access on 16 November 2025) and Pma_NU_1.0: https://ftp.ncbi.nlm.nih.gov/genomes/all/GCF/040/436/345/GCF_040436345.1_Pma_NU_1.0/, access on 16 November 2025) have been released publicly, both exhibit certain limitations in *P. major*. For instance, Pmaj_1.0 lacks robust chromosome-level scaffolding, while Pma_NU_1.0 lacks detailed genetic background information (e.g., sex), thereby constraining studies. In this study, a chromosome-level genome of a male *P. major* that served as the heterospecific paternity for gynogenetic *P. olivaceus* was assembled, providing a new resource to investigate the allo-sperm effect in marine fishes. The final assembly genomic size was 795.23 Mb, with a contig N50 of 11.22 Mb, scaffold N50 of 32.03 Mb, and a chromosome anchoring rate of 95.44%. Relative to Pma_NU_1.0, Contig N50 is markedly improved (11.22 Mb vs. 8.7 Mb) and the total assembly size is 9.23 Mb larger, offering a more complete and accurate genomic resource for future breeding, evolutionary and comparative studies.

Gynogenesis is traditionally recognized as a specialized form of unisexual reproduction in which paternal sperm activates the egg but does not contribute genetically to the embryo. As a result, the offspring inherit their entire genetic material from the maternal genome. This reproductive technique has been widely applied in aquaculture to produce all-female populations and for genetic improvement [68]. For the first time, satellite DNA fragments were identified as paternal genetic markers in gynogenetic grass carp (grass carp × koi carp), providing evidence for the existence of an allo-sperm effect [69,70]. Further studies demonstrated paternal DNA retention in offspring when sperm from different species were used to activate gynogenesis, resulting in phenotypic variation (*Carassius auratus var. Pengsenensis* (♀) × *Elopichthys bambusa* (♂) and *Carassius auratus var. Pengsenensis* (♀) × *Culter alburnus* (♂)) [9]. In the present study, artificial gynogenesis in *P. olivaceus* was induced using UV-irradiated sperm from *P. major*. Paternal-specific reads mapped to *P. major* revealed eight representative paternal-specific DNA sequences in the gynogenetic offspring of *P. olivaceus* produced using UV-irradiated *P. major* sperm. These sequences showed complete query coverage (100%) and high nucleotide identity (>96.88%) with all *P. major* representatives, indicating an allo-sperm effect in gynogenetic *P. olivaceus*. While natural polyploidy and gynogenesis have been widely documented in freshwater fishes, analogous cases in marine fish species are rare, with the exception of Salmoniformes, likely due to their anadromous life history strategy [71]. This suggests that marine fish may possess more stringent mechanisms for maintaining genomic stability compared to freshwater species. Notably, some freshwater fish have been reported to undergo gynogenesis without the need for UV-irradiated sperm to inactivate the paternal genome9. Recent studies have documented paternal DNA introgression has been documented in closely related species: Grass carp (♀) × Koi carp (♂) [69,70], allodiploid blunt snout bream (♀) × topmouth culter (♂) [72], allotetraploid red crucian carp (♀) × common carp (♂) [73], autotetraploid red crucian carp (♀) × blunt snout bream (♂) [74]. Remarkably, a trans-order allo-sperm effect has been demonstrated between *P. olivaceus* (Pleuronectiformes) and *P. major* (Spariformes), suggesting that the allo-sperm effect can occur even between distantly related taxa.

The centromere, a specialized chromosomal structure composed of tandemly repeated satellite DNA, is crucial for sister chromatid cohesion and accurate chromosome segregation during meiosis and mitosis [75,76]. Centromeric DNA sequences are highly species-specific, with conservation typically limited to closely related species [77]. In gynogenetic *P. olivaceus*, centromeric repeat sequences derived from *P. major* were identified, suggesting paternal DNA transmission through gynogenetic inheritance. Previous research has shown that the EcoRI satellite DNA family constitutes a conserved centromeric component among Sparidae species [64]. This family is defined by the consensus motif (A/T)CTGAAA(A/C)(G/C) [64,78]. The sequence contains four conserved loci at positions 11–19, 47–55, 68–76, and 147–155 in *Diplodus sargus*, *Diplodus annularis*, *Diplodus puntazzo*, *Diplodus bellottii*, *Lithognathus mormyrus*, *Spondyliosoma cantharus*, and *S. aurata*. In contrast, *Pagellus erythrinus* exhibits this motif only at positions 11–19, which is consistent with the structure of the paternal-specific sequences identified in *P. major*-derived gynogenetic offspring (Figure 3). Thus, the EcoRI satellite DNA sequence may serve as an effective molecular marker for distinguishing meiotic gynogenetic individuals from normal diploids in *P. olivaceus*.

Although centromeres perform a conserved function across eukaryotes, their satellite DNA sequences evolve rapidly and show significant interspecific variability [79,80]. In this study, centromeric satellite sequences from *P. major* were detected in offspring generated by both mitotic and meiotic gynogenesis. However, the meiotic gynogenetic offspring exhibited longer and more uniform *P. major* centromeric satellite sequences, suggesting differences in genomic stability between the two gynogenetic methods. In meiotic gynogenesis, eggs are cold-shocked (0 °C for 45 min) to inhibit extrusion of the 2nd polar body, 3 min after insemination with UV-irradiated sperm. The UV-irradiated sperm nucleus becomes condensed, measuring approximately 1.7 μm in diameter at the metaphase of the first mitosis [81]. For mitotic gynogenesis, eggs are incubated at 17 °C for 60 min post-insemination, followed by pressure shock. Here, the condensed sperm nucleus measures about 3.2 μm in diameter at metaphase [82]. In both processes, condensed sperm are positioned on the equatorial plate of the bipolar spindle. The extended cold-shock treatment in meiotic gynogenesis likely inhibits sperm nucleus enlargement, potentially influencing both the size and type of inserted centromeric satellite sequences [8,81,82].

## 5. Conclusions

A high-quality, chromosome-level assembly of the *P*. *major* genome was presented, integrating Illumina short-read, PacBio HiFi long-read, and Hi-C data. Comparative analyses revealed 75 gene families that are significantly expanded in clades with reduced sperm size, implicating potential regulatory role in spermiogenesis and sperm structural specialization. Eight paternal-specific fragments (>96.88% identity) were uniquely recovered from gynogenetic *P*. *olivaceus*, providing unambiguous evidence of an allo-sperm effect in marine teleosts. Our findings extend the observation of the allo-sperm effect to marine fish species and demonstrate its potential to occur across taxonomically distant taxa, as evidenced by the cross between *P. olivaceus* (Pleuronectiformes) and *P. major* (Spariformes). Key achievements not only enrich the genomic information available for *P. major* but also offer significant insights for precision breeding and marine evolutionary biology.

## Figures and Tables

**Table 1:** Statistics of *P. major* sequencing data and genome assembly.

Item	Category	Number
Sequencing Data	Pacbio HiFi (Gb)	22.72
	Illumina WGS (Gb)	43.09
	Hi-C (Gb)	89.32
Survey	Estimated genome size (Mb)	772.32
	Heterozygosity	0.65%
Assembly	Assembled genome size (Mb)	795.23
	Contig number	362
	Contig N50 (Mb)	11.22
	Contig N90 (Mb)	3.67
	Largest contig (Mb)	28.56
	Scaffold number	479
	Scaffold N50 (Mb)	32.03
	Scaffold N90 (Mb)	23.69
	Largest scaffold (Mb)	39.52
	Anchoring rate	95.44%

**Table 2:** Summary statistics of WGS and mapping reads.

	Raw Data (G)	Clean Data (G)	Depth (×)	Mapping Rate (*P. olivaceus*) (%)	Mapping Rate (*P. major*) (%)	*p*-Value (vs. Nor_fd)
Mit_gd	6.97 ± 0.65	6.91 ± 0.65	11.75 ± 1.11	99.57 ± 0.22	20.48 ± 12.65	0.000010
Mei_gd	6.58 ± 0.28	6.53 ± 0.28	11.10 ± 0.48	99.69 ± 0.04	18.60 ± 3.00	0.072492
Nor_fd	6.60 ± 0.23	6.54 ± 0.23	11.12 ± 0.39	99.63 ± 0.07	12.12 ± 2.03	-

## Data Availability

The raw sequence reads are deposited into the National Genomics Data Center (NGDC) database with the GSA No. of CRA025535. The genome assembly is deposited into the NGDC database with the BioProject No. of PRJCA039835. The 90 resequencing data of *Paralichthys olivaceus* originated from previous research have been deposited into the NGDC database with the GSA No. of CRA018591.

## Supplementary Materials

The following supporting information can be downloaded at: https://www.mdpi.com/article/10.3390/biom15121648/s1, Supplementary File S1: Sanger sequencing chromatogram.

## Abbreviations

The following abbreviations are used in this manuscript:

BUSCO	Benchmarking universal single-copy orthologs
LINE	Long interspersed nuclear elements
SINE	Short interspersed nuclear elements
LTR	Long terminal repeat
Bp	Base pairs
MYA	Millions of years ago
Gb	Gigabases
Mit_gd	Mitotic gynogenetic diploids
Mei_gd	Meiotic gynogenetic diploids
Nor_fd	Meiotic gynogenetic diploids
ad-Mit_gd	Additional mitotic gynogenetic diploids
ad-Mei_gd	Additional meiotic gynogenetic diploids
NCBI	National center for biotechnology information
KEGG	Kyoto encyclopedia of genes and genomes

## Appendix A

**Table A1:** Primers used in validation of paternal-specific sequences.

Primer Names	Primer Sequences (5′→3′)		Amplification Product Size
	Forward	Reverse	
G441-primers	TCATTAAAGGTGCAAACTGGC	GACCCGAATTCAACCAGTTCA	150
G608-primers	CGGCTAAGCTCCATCACCTA	CCCTCTGCTTCAAAGGTCCT	234
G462-primers	GCTACGTCACTTCCGGTTTC	AACACTTGGGCGGATTTCAC	205

**Table A2:** Transposable elements statistics in *P. major.*

Type	Repbase TEs		De Novo		Combined TEs	
	Length (bp)	% in Genome	Length (bp)	% in Genome	Length (bp)	% in Genome
DNA	21,097,913	2.65	52,107,479	6.55	73,205,392	9.20
LINE	16,163,963	2.03	19,942,205	2.51	36,106,168	4.54
SINE	1,443,154	0.18	1,080,688	0.14	2,523,842	0.32
LTR	9,182,293	1.15	10,169,888	1.28	19,352,181	2.43
Other	24,623,917	3.10	5,144,005	0.65	29,767,922	3.74
Unknown	336,642	0.04	84,933,826	10.68	85,270,468	10.72
Total	72,847,882	9.16	173,378,091	21.80	246,225,973	30.96

**Table A3:** The comparison of statistics of the gene structure across different species.

Species	Predicted Protein-Coding Number	Average Gene Length (bp)	Average Exon Length (bp)	Average Exon Number	Average Intron Length (bp)	Average Intron Number
*Pagrus major*	29,083	13,821.71	183.32	8.79	1566.45	7.79
*Sparus aurata*	25,222	20,229.04	232.87	9.57	1814.80	8.57
*Acanthopagrus latus*	23,810	19,831.93	301.93	11.65	1553.45	10.60
*Paralichthys olivaceus*	23,126	14,847.16	262.87	10.33	1296.53	9.32
*Takifugu rubripes*	21,411	11,809.09	221.10	9.66	923.29	8.66

**Table A4:** Statistics of the annotated protein-coding genes in *P. major.*

Type		Number	Percent (%)
Annotation	TrEMBL	24,804	85.29
	Swiss-Prot	19,904	68.44
	EggNOG	23,196	79.76
	NR	25,108	86.33
	InterPro	26,624	91.54
Total	Annotation	27,474	94.47
	Gene	29,083	100.00

**Table A5:** Statistics of Sperm Structure in Fish.

Species	Average Diameter of the Longitudinal Section (μm)	Average Diameter of the Transverse Section (μm)	Reference
*Danio rerio*	2.93	2.93	[52]
*Colossoma macropomum*	2.74	2.74	[53]
*Ictalurus punctatus*	2.30	2.30	[55]
*Pagrus major*	1.88	1.88	[44]
*Sparus aurata*	1.87	1.87	[45]
*Scophthalmus maximus*	1.74	1.74	[48]
*Paralichthys olivaceus*	1.49	1.49	[47]
*Salmo salar*	2.93	2.43	[50]
*Oncorhynchus kisutch*	2.39	1.86	[51]
*Siniperca chuatsi*	1.43	1.02	[54]
*Cynoglossus semilaevis*	1.36	0.87	[49]
*Takifugu rubripes*	1.35	0.65	[46]

**Table A6:** Summary statistics of Contig N50 and number by distinct software.

	SOAPdenovo2			SPAdes			MEGAHIT		
	Contig N50	Contig Number	Summary	Contig N50	Contig Number	Summary	Contig N50	Contig Number	Summary
Mit_gd	242.00	242.00	160,480.87	426.53	205.83	74,004.20	524.27	183.90	93,575.97
Mei_gd	258.70	287.53	59,974.43	436.63	74.40	31,751.87	456.97	111.70	47,468.63
Nor_fd	264.57	174.03	47,158.70	413.27	64.53	27,342.13	484.93	91.87	41,810.50
